# Deep learning for studying drawing behavior: A review

**DOI:** 10.3389/fpsyg.2023.992541

**Published:** 2023-02-08

**Authors:** Benjamin Beltzung, Marie Pelé, Julien P. Renoult, Cédric Sueur

**Affiliations:** ^1^CNRS, IPHC UMR, Université de Strasbourg, Strasbourg, France; ^2^ANTHROPO LAB – ETHICS EA 7446, Université Catholique de Lille, Lille, France; ^3^CEFE, Univ Montpellier, CNRS, EPHE, IRD, Montpellier, France; ^4^Institut Universitaire de France, Paris, France

**Keywords:** deep learning – artificial neural network, drawing behavior, sketch, artificial intelligence – AI, art cognition, primates

## Abstract

In recent years, computer science has made major advances in understanding drawing behavior. Artificial intelligence, and more precisely deep learning, has displayed unprecedented performance in the automatic recognition and classification of large databases of sketches and drawings collected through touchpad devices. Although deep learning can perform these tasks with high accuracy, the way they are performed by the algorithms remains largely unexplored. Improving the interpretability of deep neural networks is a very active research area, with promising recent advances in understanding human cognition. Deep learning thus offers a powerful framework to study drawing behavior and the underlying cognitive processes, particularly in children and non-human animals, on whom knowledge is incomplete. In this literature review, we first explore the history of deep learning as applied to the study of drawing along with the main discoveries in this area, while proposing open challenges. Second, multiple ideas are discussed to understand the inherent structure of deep learning models. A non-exhaustive list of drawing datasets relevant to deep learning approaches is further provided. Finally, the potential benefits of coupling deep learning with comparative cultural analyses are discussed.

## Introduction

Drawing is a powerful communication medium that can convey concepts beyond words. Two different approaches are traditionally used to study drawing behavior (Pysal et al., 2021): the process approach (Freeman and Cox, 1985; Adi-Japha et al., 1998) and the product approach (Brooks, 2009; Xu et al., 2009). The process approach analyzes drawings through the behavioral characteristics linked to the drawing task and the individual who is drawing. For example, this perspective may require information on behavioral sequences (investigated through coordinates and the time spent drawing each point or behavioral sampling), which is more difficult to collect than the data needed for the product approach. Indeed, the latter analyzes the result of the drawing, based only on spatial and visual information, to infer the underlying behavior. Drawings, as final products, have been widely used to better understand the cognitive capacities of individuals, in particular to investigate the cognitive development of children (Malchiodi, 1998; Barraza, 1999; Cox, 2005; Farokhi and Hashemi, 2011). Studying visual features such as the color palette in drawings, the product approach has been pivotal in describing the diversity of personalities in children (Goldner and Scharf, 2011), identifying mental disorders (Tharinger and Stark, 1990) and post-traumatic symptoms (Backos and Samuelson, 2017), and even revealing concealed emotions (Fury, 1996). Both of these approaches – process and product – are covered in this review.

In toddlers, first drawings are in the form of scribbles, described as a motor activity not directed by the eyes, but by the mechanical functioning of the motor system arm-wrist-hand (Piaget and Inhelder, 1967; Freeman, 1993). At this age, scribblers appear to take little interest in their final products, whereby the process of drawing itself or improving the technique prevails over the will of representation (Thomas and Silk, 1990; Golomb, 1992). Figurative drawings, where what is drawn is representative for both the subject and external eyes, only appear at 3–4 years of age (Golomb, 1992; Freeman, 1993).

However, figuration and internal representativeness are not always similar. Since the end of the 19th century, researchers have developed a methodology to address the difficulties of studying drawings and scribbles (Farokhi and Hashemi, 2011). These analyses are limited by the subjective judgment of the observer (Lark-Horovitz, 1942), which is prone to several biases, especially with respect to semantic analyses. These issues are minor when computing low-level features such as color statistics, but are fundamental when trying to extract higher-level features; for example, one observer may see a house where another observer only sees a scribble, or both observers may fail to detect the drawer’s intention to represent a house. The distinction between figuration and internal representativeness is essential, particularly when analyzing young children’s drawings. Indeed, while previous theories proposed that the drawing among the youngest reflect motor activity only, recent studies have provided evidence for a symbolic function of drawing as early as 2 years old, suggesting that even young children can learn and become aware of the two visual aspects of drawing: the referent, which is the concept of what is drawn, and the signifier, which is the drawing object itself (Longobardi et al., 2015). However, a young child using drawings for symbolic representation may not intend to represent the formal aspects of reality through his or her first drawings, but rather seeks to express the world around him or her in a physiognomic way, using the line as means of expression (Longobardi et al., 2015). In other words, what is regarded as a scribble for an adult can be a symbolic representation for a young child. To understand the emergence and development of drawings, it is important to interpret such drawings. To do so, asking very young children about their product is impossible, as they cannot communicate verbally. To address this problem, one could ask adults to interpret the drawings. However, by doing so, adults would typically fail to detect the intention of the drawer and the meaning of scribbles. Asking the child about his/her intention only partially solves this problem because for a given child, the answer has been shown to vary from 1 day to the next (Martinet et al., 2021). The answer is also dependent on the subject’s verbal communication skills, which are naturally limited in toddlers, as in other great apes. This is not a problem for free-form drawings (i.e., no instruction), but becomes challenging for task-based drawings (i.e., instructions and constraints on the drawings; Martinet et al., 2021). The same problem arises among great apes such as chimpanzees (*Pan troglodytes*), who are well known for their drawing behavior (Martinet and Pelé, 2020). Indeed, captive chimpanzees spontaneously draw and paint if provided with appropriate materials (pen, paint, brushes, and paper) and can continue this behavior without being reinforced with food (Boysen et al., 1987; Tanaka et al., 2003).

To interpret the intention behind drawings, objective and mathematical analyses have been developed. Martinet et al. (2021) elaborated an innovative mathematical tool based on spatial fractal analysis, and Beltzung et al. (2021) used temporal fractal analysis for this purpose. The combination using a principal component analysis of simple metrics (number of lines, circles, colors, cover rate, etc.) can also provide interesting results regarding interindividual differences in human (Sueur et al., 2021) or orangutan drawings (Pelé et al., 2021).

### The rise of deep learning

Over the last few decades, researchers have been investigating drawings using AI and computer vision (Eitz et al., 2012; Li et al., 2013). The latter encompasses sophisticated techniques and algorithms which can extract features in an image that are meaningful to human visual perception, such as facial features (e.g., eyes and nose). These techniques are widely used for detection [e.g., corner and edge detection (Li Y. et al., 2015)], segmentation (e.g., K-mean, P-Tile), and recognition (convolutional neural network). Most analyses use computer vision to extract features which are then fed into a classifier.

It is important to note that traditional models and machine learning have been successfully used as approaches to study the drawing behavior. For example, by measuring the proportion of time the pen was in contact with the paper, Cohen et al. (2014) have shown a link between the Digital Clock-Drawing test and depression. Polsley et al. (2021) used machine learning methods, as Random Trees and Random Forest, to demonstrate how curvature and corners in drawings are linked to the age. These mathematical analyses and indices are objective contrary to former measures and are a good starting point for developing more objective studies using artificial intelligence (AI).

**Figure 1:**
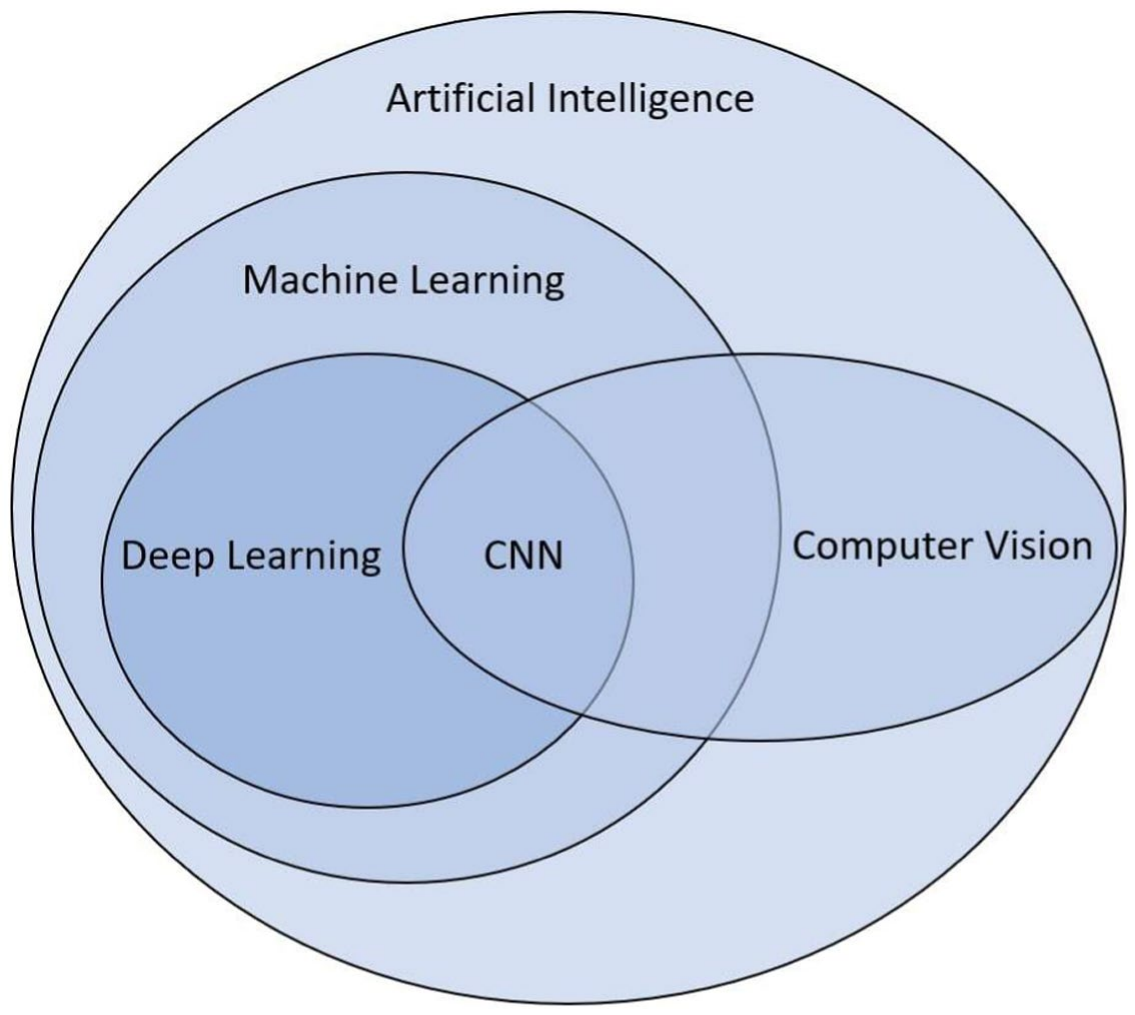
Euler diagram of artificial intelligence and neural networks in computer vision.

Currently, the most efficient and promising way to learn from images, including drawings, is deep learning (Figure 1; Ravindran, 2022), a sub-branch of computer vision and artificial intelligence, and more precisely neural networks, also used for speech recognition (Graves et al., 2013) and text classification (Liu et al., 2017). Deep learning allows us to go further by avoiding some anthropomorphic biases, such as the confirmation bias. For example, when analyzing drawings without deep learning, the features may be unconsciously selected accordingly to the beliefs of the human devising this process. By using almost raw data, deep learning thus reduces such biases.

**Figure 2:**
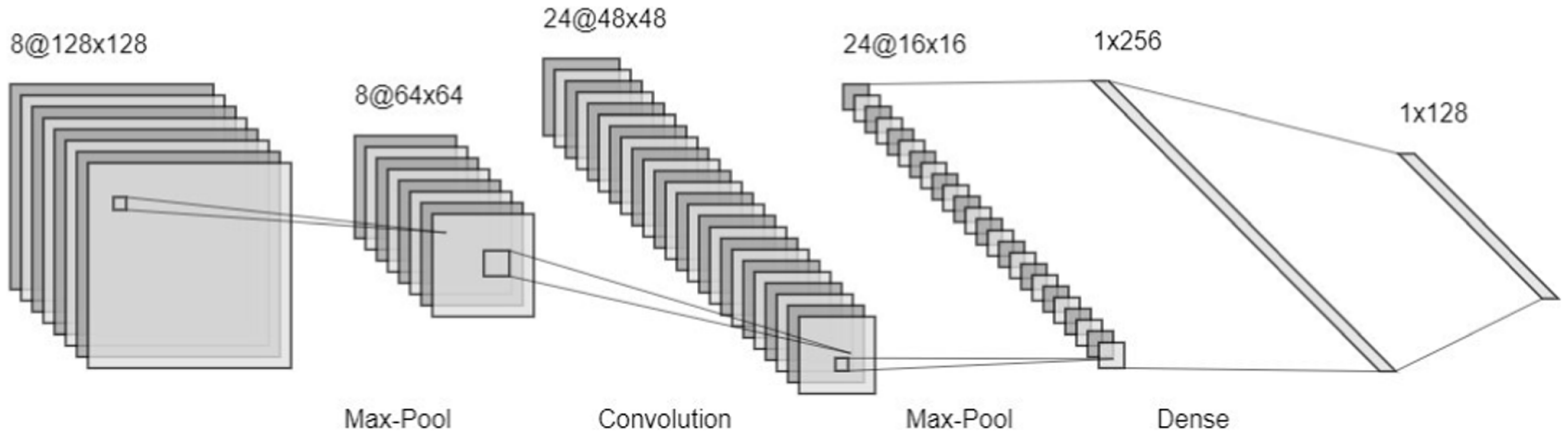
Example of a CNN architecture. The model takes an image as input; the image passes through layers to finally be classified between predefined classes.

The first mathematical model defining the concept of artificial neurons dates back to McCulloch and Pitts (1943). Deep learning only surged in 2012, when a deep convolutional neural network (CNN) named *AlexNet* (Krizhevsky et al., 2012), outperformed other methods by a large margin in a popular competition of image classification, the ImageNet Large Scale Visual Recognition Challenge (ILSVRC; Deng et al., 2009). CNNs (Figure 2) form a subcategory of artificial neural networks, specifically designed for processing images by learning filters (*via* convolutional layers) that optimize performance in a predefined task (e.g., categorizing images or regressing images with a continuous variable). These filters allow capturing a hidden representation of images (Mukherjee and Rogers, 2020). A glossary of technical terms is presented in Table 1.

**Table 1:** Glossary of technical terms.

Terms	Meaning	Definition
AI	Artificial Intelligence	All techniques allowing reproduction of intelligence.
ML	Machine Learning	Subset of AI techniques which learn from the training data.
DL	Deep Learning	Subset of ML techniques based on artificial neural networks. The analyzed features are learned by the model.
ANN	Artificial Neural Network	Model consisting of layers made up of units, also called neurons. An ANN can be shallow, or deep (DNN) when consisting of at least 2 hidden layers (i.e., layers between input and output).
CNN	Convolutional Neural Network	An ANN is specifically designed for images using convolutional layers.
RNN	Recurrent Neural Network	An ANN designed to process sequences, such as time series.
GNN	Graph Neural Network	An ANN where node relationships are studied.
GAN	Generative Adversarial Network	Unsupervised DL method capable of generating fake but realistic data.
VAE	Variational Autoencoder	An ANN belonging to the family of autoencoders, consisting of an encoder that compresses the data, and a decoder that reconstructs the data. Reconstruction in VAE is through a sampling of the hidden representation of the statistical model, rather than the hidden representation itself.
DA	Data augmentation	Techniques allowing an increase in the number of training data, by altering them through different transformations.
TL	Transfer Learning	Method consisting of reusing a model already trained on another task.

Although deep learning is now a flagship approach to image analysis, most of these algorithms have been trained and designed for photos. Compared to photos, drawings and sketches are sparser and can be abstract. DL models thus need to be created to specifically process this type of data (Zhang et al., 2016; Yu et al., 2017; Pysal et al., 2021).

These models can successfully classify drawings from several categories with high accuracy but allow limited interpretability. Indeed, deep learning models are often considered black boxes because of the number of parameters reaching tens of millions (Krizhevsky et al., 2012). Nevertheless, as in all scientific domains, interpretability and comprehension are key points when developing a model. What does a model outperforming human recognition ‘discover’ and ‘comprehend’ in the data that humans do not? Is it possible to extract and decipher the discriminant features and are humans able to understand them? To improve the interpretability of these models and to answer these questions, multiple methods have been developed and are discussed later in this review. Nevertheless, interpretability and explainability remain important challenges in deep learning (Gilpin et al., 2018) and are among the most active research topics in AI (Zhang et al., 2016; Wu T. et al., 2018; Rudin, 2019). According to Gilpin et al. (2018), “the goal of *interpretability* is to describe the internals of a system in a way that is understandable to humans” and *explainability* (for deep networks) consists in giving an explanation to “the processing of data inside a network, or explaining the representation of data inside a network” (note that the definitions of these concepts are still debated, see for example Tjoa and Guan, 2021). When studying drawings, the interpretability of AI is also fundamental to improving the knowledge of the ontogeny of drawing and the emergence of representativeness. Likewise, the AI processing of children and chimpanzees drawings can be compared to allow a better understanding of the evolutionary history of drawing. To achieve this goal, the assumptions on the underlying mechanisms of the drawing behavior can be formalized and implemented in a neural network model. With this objective, Philippsen and Nagai (2019) combined Bayesian inference and deep learning. They developed a neural network capable of completing partial drawings based on prior information. The goal of their study was to use this model to replicate children’s and chimpanzees’ drawing behavior to analyze the relative importance of different priors.

As previously mentioned, in children, the quality and representativeness of drawings improve with age (Martinet et al., 2021). In addition to age, other variables influence representation, such as sex (Picard and Boulhais, 2011) and cultural background (Alland, 1983; Gernhardt et al., 2013). For example, Gernhardt et al. (2013) demonstrated that the number of facial details and facial expressions in drawings vary among children from different cultures. Deep learning is a promising tool for understanding cultural variations in drawing. To the best of our knowledge, no such studies have been carried out yet. However, deep learning applied to drawings has recently been used to characterize mental disorders in individuals, such as Autism Spectrum Disorder (Anne et al., 2018), to predict the Draw-a-Person test (Widiyanto and Abuhasan, 2020), the Clock-Drawing test (Chen et al., 2020), and detect mild cognitive impairment (Ruengchaijatuporn et al., 2022).

Overall, deep learning in complement to other machine learning methods has the potential to greatly improve our knowledge of the ontogeny and evolutionary history of drawing behavior. This review presents and discusses the different applications of deep learning in drawing analysis and aims at giving the keys for readers who are interested by using deep learning to study drawing behavior and want to go further. The first section introduces different approaches to drawing analysis based on deep learning, which have already been applied or appear promising. These approaches are not discussed in relation to their performance (e.g., score of accuracy), but on the insights they can bring on the understanding of the drawing behavior. The second section reviews publicly available datasets that are well suited for studying drawings and sketches using AI and outlines the challenges. The review is concluded by discussing future research frameworks and perspectives in deep learning as applied to drawings.

## Approaches in deep learning for drawing(s) analysis

This section is divided into two parts. The first part is focused on model-centric analyses, which refers to studies directly using the outputs of a model to make interpretation of the results. The second part focuses on analyses based on model-internals. The studies considered in this part use the weights of a model after being trained (i.e., *post hoc* interpretation methods), such as heatmaps, to discover for example of the information is encoded in the model.

### Model-centric analyses

As in classical drawing studies, deep learning approaches can be classified as focusing either on drawings as a product or on the process of generating drawings and sketches. While the first approach investigates only the spatial dimension of drawings, the second considers the temporal dimension.

#### Product approach

##### Prediction-based analyses

Machine learning models are often trained with the aim to predict labels for unlabeled data (i.e., that have never been seen by the model). In deep learning, this prediction task can be conducted at several levels, from labeling the image as a whole (classification) or predicting a label for every pixel of an image (segmentation).

###### Classification

The most popular application of deep learning is classification. Classification plays a major role in computer vision in tasks as varied as classifying Alzheimer’s disease from magnetic resonance images (Wen et al., 2020), identifying fish species (Li X. et al., 2015), and recognizing malware images (Yue, 2017). Classification is also a preliminary step in other tasks, such as segmentation. CNNs are mostly used for image classification in a supervised learning paradigm, where a model is trained to classify images into categories predefined by the user, by learning from a dataset of labeled images (i.e., images for which the category is known). Once trained, the model is used to predict the categories of new, unlabeled images.

The first CNN developed for sketch classification was Sketch-a-Net (Yu et al., 2017), which achieved better performance than humans in object classification. It may be surprising that a model trained on data labeled by humans can outperform humans at classification. Indeed, CNNs learn a latent representation, that is, hidden features from the data, which is more complex than human representation. Sketch-a-Net performs better on sketches than neural networks trained on photos, highlighting the need for specific architectures for drawings (Yu et al., 2017). A CNN can thus outperform and replace classical methods used in sketch classification based on predefined classes. For example, in psychology, Vaitkevičius (2019), built a CNN capable of successfully classifying scribbles in 20 different classes as defined by the psychologist Rhoda Kellogg (e.g., “single dot,” “imperfect circle through single vertical line,” “spiral line”). Compared to other classifiers used in computer vision (e.g., support vector machine (SVM), random forest, k-nearest neighbors), CNN achieves the best results, matching the efficiency of neural networks in analyzing non-figurative drawings, demonstrating how deep learning can automatize complex and laborious tasks. Another example is Rakhmanov et al. (2020), who used a simple CNN architecture (two convolution layers and two fully connected layers) to classify drawings according to the Draw-a-Person test (Goodenough, 1926), a cognitive test in which the subject (a child, most often) draws a human figure, and a score is assigned to the drawing based on several criteria (e.g., the number of eyes, body proportions, presence of the mouth) to assess the child’s intellectual maturity. This test is used for several purposes, such as detecting behavioral disorders or measuring nonverbal intelligence. Several parameters are tested during the training of the neural network mode, and data augmentation is applied to compensate for the low number of drawings, to significantly increase the accuracy. Data augmentation is a computer vision technique which is widely used in machine learning, which increases the size of the training data set by slightly modifying the original instances (that are images in this case, by applying rotation, horizontal flip, color contrasts for example) during the training phase. DA also reduces overfitting, a phenomenon that occurs when the model is too specialized for the training data and generalizes poorly on new data. Although the deep learning model was able to learn and produce relevant results, Rakhmanov et al. (2020) found that other methods of computer vision, such as the bag of visual words (BoVW) approach, outperformed CNN (62% accuracy for BoVW versus 52% for CNN). This example shows that a straightforward CNN design does not necessarily outperform state-of-the-art methods.

Thus, more complex CNN structures are required to learn the hidden representation of an object from sketches. To this end, Zhang et al. (2016) built *SketchNet*, a neural network capable of classifying sketches in object categories to discover the shared structures between real images and sketches belonging to the same category. The classification part of this model relies on associating a sketch image with a positive real image (from the same category) and a negative image (from another predicted category). The authors used an architecture consisting of three subnetworks optimized to extract features of the sketch images, positive images, and negative images. The features of the sketch and real images were eventually merged. *SketchNet* is based on prediction rankings. For a given sketch, the model computes the probability of the sketch belonging to each category, before returning the top five prediction categories (i.e., the five more likely predicted classes for this sketch) and the nearest real images.

###### Segmentation

**Figure 3:**
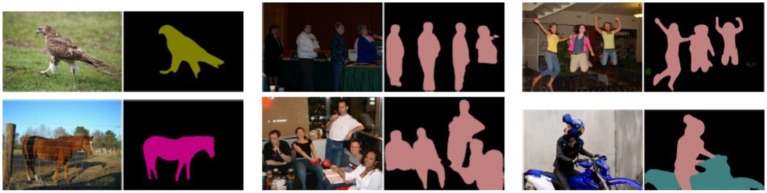
Examples of segmentation results through CNNs from Chen et al. (2017). Reprinted with permission.

Classification can be used as a preliminary step for other tasks, such as segmentation. Image segmentation partitions the pixels of an image into multiple regions and assigns a label to each pixel. This technique is widely used in various fields, such as medicine (Brzakovic and Neskovic, 1993) and video surveillance (Patro, 2014). It can rely on classical computer approaches, but more recently, also on deep learning (Figure 3).

While classification helps improve segmentation, the opposite is also true. Sketches can be classified as a whole after segmentation and analysis of individual components, as in semantic sketch segmentation (SSS), which aims at labeling individual strokes. Semantic segmentation is notoriously difficult, however, because of complex perceptual laws, such as those proposed by Gestalt theory (Wertheimer, 1938). For example, the law of closure states that in an image with missing parts, the brain visually fills in the gaps. Interestingly, CNNs have been found to reproduce some perceptual laws. It appears that perceptual laws may or may not be present depending on the training set, and more generally the weights of the model (Kim et al., 2019, 2021; Jacob et al., 2021). For these reasons, it is a complex task to understand if and how neural network perception differs from that of humans, and these questions are still debated. Moreover, as with classification, it is necessary to develop architectures and models of semantic segmentation for sketches, specifically because of the differences between sketches and photos.

One of the first CNN-based models of sketch segmentation was *SketchParse,* proposed by Sarvadevabhatla et al. (2017). *SketchParse* automatically parses regions of sketches and has proven to be effective, for example, in separating the head, body, and tail of a horse. However, *SketchParse* parses regions, not strokes, which limits the utility of segmentation in studying drawings as a process because regions most often are not consistent with strokes.

Graph neural networks (GNNs) can overcome this limitation. Starting from these neural networks, it is possible to cluster strokes into semantic object parts. Yang et al. (2021) proposed *SketchGNN*, a convolutional GNN which outperforms state-of-the-art models, such as SSS and stroke labeling. Their model also extracts features at three different scales: point-level, stroke-level, and sketch-level. *SketchGNN* can for example label each pixel of a sketch representing a face, to associate with the pixel a larger face component, such as the nose or the mouth, without taking into account the order of the strokes. Predicting object parts by strokes labeling could allow for comparing the structure of specific parts of an object depending on the culture of the drawers, for example to compare object proportions. Another interesting SSS model was proposed by Li et al. (2019). Their model is an hourglass-shaped network consisting of an encoder and a decoder. The 2D image passes through a network which predicts the segmentation map. The corresponding segmentation map is then transformed into a stroke-based representation, which is used to refine the segmentation map. Due to the lack of 2D annotated sketches, the network is trained on edge maps extracted from 3D models already segmented and labeled, which can thus be transformed into sketches. Moreover, as the model is trained on 3D models, several viewpoints are available, that may not be the ones frequently represented in drawings. As it would be questionable to analyze freehand sketches by using a network mainly trained on 3D model-transformed sketches, the authors evaluated their model on freehand sketch datasets (Eitz et al., 2012; Huang et al., 2014); their model outperformed previous ones. Comparing children’s and 3D model-transformed sketches, for a given category, could improve our knowledge of their spatial representation.

##### Generation-based analyses

Deep learning used for image classification and segmentation is usually referred to as discriminative AI, where models are trained to convert high-dimensional inputs (e.g., images) into low-dimensional outputs (e.g., the names of depicted objects). In contrast, generative AI generates high-dimensional outputs (e.g., images) from low-dimensional inputs (e.g., semantic representations). Most people know generative AI through web-based applications that allow drawing one’s portrait in Van Gogh’s style or putting fake words in Obama’s mouth on a video. However, beyond these applications, generative AI has become one of the most growing research areas in AI because of a very large array of applications (Wu et al., 2016; Kell et al., 2020; Yang and Lerch, 2020; Bian and Xie, 2021), which include a study of drawings.

###### Generation

Pallavi (2019) devised SuggestiveGAN, a generative adversarial network (GAN; Karras et al., 2019). A GAN is an unsupervised algorithm in which two neural networks compete. Fundamentally, one neural network (the discriminator) is a classifier to distinguish real images from fake images; the other neural network (the generator) tries to generate the most ‘realistic’ fake images (according to the real dataset). SuggestiveGAN is able to reconstruct incomplete drawings (with missing strokes). The proposed model grasps the structure of the drawings at the expense of the details.

###### Style transfer

Style transfer involves applying the style of an image to another image, but not the content. Gatys et al. (2016) proposed a CNN-based method of style transfer that quickly achieved high popularity owing to its impressive visual results. The method has been popularized by the famous Van Gogh painting, whose style has been widely transferred onto various kinds of portraits and landscape photos. The authors defined the style from the Gram matrix of activations, a measure of covariation between filters within a given layer (usually, all convolutional layers are used to define the style). The content is defined by the activation of the deepest convolutional layers. The stylized image is then obtained by searching a new image that simultaneously minimizes the distance between its content and that of the ‘content’ image, and the distance between its style and that of the ‘style’ image. One of the most famous examples of Gatys et al. (2016) model is the transfer of Van Gogh’s painting style to photographic portraits or landscapes.

Since the seminal work of Gatys et al. (2016), other CNN-based style transfer algorithms have been proposed and applied to various contexts [e.g., in user-assisted creation tools (Jing et al., 2020)]. For drawings and sketches, it is necessary to design specific models of style transfer as in the classification of drawings which are sparser and have a higher level of abstraction compared to paintings. Chen Y. et al. (2018) proposed *CartoonGAN*, a GAN-based style transfer algorithm developed for cartoon stylization. The model generates cartoon images based on real-world photos, which can be useful for photo editing or for artists to gain time. More recently, Chen C. et al. (2018) proposed a framework capable of synthesizing face sketches while preserving details, such as skin texture and shading.

Hicsonmez et al. (2017) applied style transfer to drawings to learn the styles of different book illustrators. Their objective was to apply the style of drawing from an illustrator (the “style image”), to an image produced by a different illustrator (the “content image”). Their framework shows that this technique can be successfully applied to drawings. Dissociating the style and content of a drawing, and modeling how these two components vary separately would have numerous implications in drawing studies. For example, by using the style of children’s drawings, one may analyze the development of motor skills through the complexity of the strokes, by using only the style component of the drawing, while dissociating the motor constraints from the representational constraints. The style component can also be used to investigate the link between different types of curves used (broken curves and smooth curves) and internal representativeness (Adi-Japha et al., 1998). Moreover, studying the development of the style of the drawing system and the writing system, using style transfer, would help in understanding the differences and similarities between the two systems. Finally, using generative AI like the one developed by Chen C. et al. (2018), but instead generating realistic photos from drawings would shed light onto children’s representation of the world.

#### Process approach

##### Prediction-based analyses

###### Classification

In addition to the product approach, sketch recognition could allow a better understanding of the cognitive processes underlying the drawing. It is known that the development of drawing and writing shows kinematic differences according to age (Adi-Japha and Freeman, 2001). Thus, classifying drawings and writing across ages could lead to discriminant low-level features, such as shapes, that could help in understanding the links as well as differences among techniques between these systems. Writing is not the only phenomenon correlated with drawings. Indeed, as shown by Panesi and Morra (2021), executive functions (e.g., shifting, inhibition) and language are linked to drawing behavior. Their work proposes several tasks to which different scores are assigned, such as the absence/presence of structures in human figure drawings, which can be further automated through deep learning. All these cognitive processes are directly linked to cortical activity, which is typically investigated using brain imaging [e.g., electroencephalography (EEG) and electromyography (EMG)]. Applying deep learning to brain imaging can be achieved within a framework such as that proposed by Leandri et al. (2021) through recurrent neural networks (RNNs), which are specifically designed for temporal sequences.

He et al. (2017) developed a model able to use the temporal information of the strokes to perform sketch recognition as well as Sketch-based Image Retrieval (SBIR), which aims at finding real images visually similar to a given sketch. The proposed model is based on a CNN coupled with a R-LSTM (Residual Long Short-Term Memory) network. Multiple representations of the drawings are learnt by considering 60, 80, and 100% of the strokes of the drawings separately. The performance achieved by this model demonstrates how stroke ordering information can be used in deep learning and how it plays a role in classification. To go further, Xu et al. (2022) proposed to consider drawings as graphs thanks to GNNs (Graph Neural Network). A classical application of such architectures is node classification. A graph consists of edges and links, and GNNs analyze the relationships between the nodes. In sketches, these types of models take the relationships between the strokes into account. Their proposed model, called Multi-Graph Transformer, allows for capturing geometric and temporal information about the drawings, as well as understanding the relationship between strokes. These models could thus be useful to improve our knowledge on the links between object parts and object representations. These approaches could also help at understand which strokes are the most relevant for classification or comparing which parts of an object are drawn first depending on the culture or age for example.

###### Segmentation

The information contained in the stroke order and temporal sequences can provide very rich information, which may be hard to decipher just through image classification. Wu X. et al. (2018) designed a stroke-level sketch segmentation model, *Sketchsegnet*, that is based on a variational autoencoder (VAE) which learns the probability distribution from the data. In *Sketchsegnet*, widely used in image generation (Razavi et al., 2019; Zhu et al., 2020), the VAE consists of a bidirectional RNN (*BiRNN*; Schuster and Paliwal, 1997) for the encoder and an autoregressive RNN (Inselberg and Dimsdale, 1990) for the decoder, thus accounting for the sequence order of strokes. For each sketch category, labels are predefined (e.g., ‘cream’ and ‘cone’ for an ice cream). Their model achieves an accuracy of 90% for stroke labeling.

Thus far, research on sketch and drawing segmentation using AI has been primarily methodological, with only rare applications to better understand the ontogeny and evolutionary history of drawing behavior. Nonetheless, segmentation could be of great interest in this kind of analysis. For example, segmentation can be used to analyze body proportions, which are indicative of the emotional state in children [e.g., disproportionally large hands can express aggression (Leo, 2015), and the relative size of the head and trunk varies with age in children (Thomas and Tsalimi, 1988)]. In addition, annotated sketch databases are not common, and annotating sketches will lead to bias, depending on the perception of the person doing the annotation. For this reason, SSS should be studied in depth through unsupervised stroke-level segmentation or by using temporal sequence algorithms (Gharghabi et al., 2019), which also consider the time spent not drawing. Applying SSS to scribbles could lead to semantic segmentation, not necessarily obvious to human perception. Moreover, SSS allows the analysis of specific regions, such as the head, at a certain level of detail despite the complexity. This could help in understanding the relative importance of different visual stimuli in shaping the representation space of children. Models using 3D sketches, similar to Li et al. (2019), can elucidate the emergence of 3D geometry in children, and more generally, the development of spatial ability in children, necessary for representativeness. Using deep learning to analyze low-level features such as the spatial distribution of strokes, their orientation and form, and how these vary with age could also be informative about the ontogeny of the drawing behavior in humans and other animals.

##### Generation-based analyses

As drawings are directly linked to the temporal sequences of the strokes, it is fundamental to consider the process when generating parts of drawings, to generate meaningful strokes. Among the first to use generative AI were Ha and Eck (2017) who studied the behavior of drawing by developing a neural network capable of reproducing and mimicking human drawing through conditional and unconditional generation. To do so, they considered each drawing as a list of points, and each point as a vector of length 5 to characterize the position and state of the pen at a given time. The generative model used in this study was VAE. In Ha and Eck’s (2017) model, both the encoder and decoder are recurrent neural networks, and hence, the name *Sketch-RNN*. When given an incomplete sketch, *Sketch-RNN* generates strokes to complete the sketch. As a result of the random nature of VAE, the model can predict different final results for the same initial sketch. The authors suggested that *Sketch-RNN* could be used, for example, to help students learn how to draw.

A model combining an RNN with Bayesian inference was developed by Philippsen and Nagai (2019) to unravel the sensory and cognitive mechanisms of drawing behavior. They relied on a ‘predictive coding’ scheme, according to which the brain constantly generates and updates internal, cognitive models of the world to predict the consequences of our actions in response to sensory inputs (Rao and Ballard, 1999). The authors investigated how varying the integration of sensory inputs with cognitive models influenced the ability of the RNN to learn to complete partial drawings. They found that a strong reliance on cognitive models is necessary to complete representational drawings, thereby highlighting the importance of internal models for efficient cognitive abilities such as abstraction and semantic categorization. Interestingly, the authors also stressed that drawings generated with a weak reliance on cognitive models differed from children’s drawings but resembled chimpanzee’s drawings. This result echoes previous suggestions that the inability of chimpanzees to complete representational drawings could be attributed to their poor predictive cognitive skills, such as those involved in imagination (Saito et al., 2010; Watanabe, 2013). This study also demonstrates the benefits of generative AI in understanding the development and evolution of drawing behavior. This predictive coding scheme can have other applications, such as understanding pathologies like metamorphopsia (e.g., straight lines that appear distorted) from the drawings of patients to unravel the neuronal mechanism that leads to these drawings.

### Model internals-based methods

As we have seen, drawing behavior can be studied by designing and training deep neural networks models and directly interpret the output. However, these approaches do not take advantage of the internal knowledge learnt by the model. To address this issue, it is possible to develop techniques that use model internals, such as the weights and the neuronal activations of each layer separately.

Predictive models based on CNNs have been shown to outperform other models such as SVM and k-nearest neighbors, in most applications. However, as with any quantitative model, predictive power comes at the cost of interpretability, and a notorious limitation of CNNs is their low explanatory appeal (Rudin, 2019). Regarding the ability of CNNs to help understand human behaviors, some researchers have suggested that AI is simply replacing a black box (the brain) with another. Other researchers have argued otherwise (Hasson et al., 2020). Ribeiro et al. (2016) developed a model to classify photographs of wolves and huskies. Based on accuracy alone, the model worked well. However, this model was in fact performing badly; all the pictures of wolves in the training set had snow in the background, and pictures of huskies did not. In learning the most discriminative features to separate images of wolves from those of huskies, the model thus focused on the presence or absence of snow in the background and did not encode the features of these canines. This purposely bad-designed experiment highlights how the qualitative analysis of learned features can increase the model interpretability. CNNs have explicit architectural specifications; they are trained with user-defined learning rules; and one has direct access to the weights (strength of connections between neurons) and neuronal activation. Analyzing how varying these hyperparameters improves or deteriorates the fit between models and empirical data offers exciting venues of research, in exploring both the neuronal mechanisms of information processing and their behavioral expressions (e.g., Richards et al., 2019; Lindsay, 2021). A remarkable example is the study by Philippsen and Nagai (2019) discussed previously, in which the authors varied the hyperparameters prior to analyzing the relative importance of sensory inputs and cognition in drawing behavior. More generally, when devising and training a model to discriminate between children and adult drawings, or between drawings of humans and other great apes, independent of model performance, one may be interested in knowing which features are responsible for AI discrimination. To do so, two possible approaches exist: local interpretation, allowing us to understand the features of a specific image (i.e., based on the data), and global interpretation, allowing us to understand class discrimination (i.e., based on the model).

#### Local interpretation

Local interpretation encompasses techniques aiming at understanding a specific prediction (i.e., for a given instance) for a given model. Applied to deep learning, such methods can help understanding which part of an image played a role for a given prediction task. This section will provide examples of such techniques applied to sketches.

**Figure 4:**
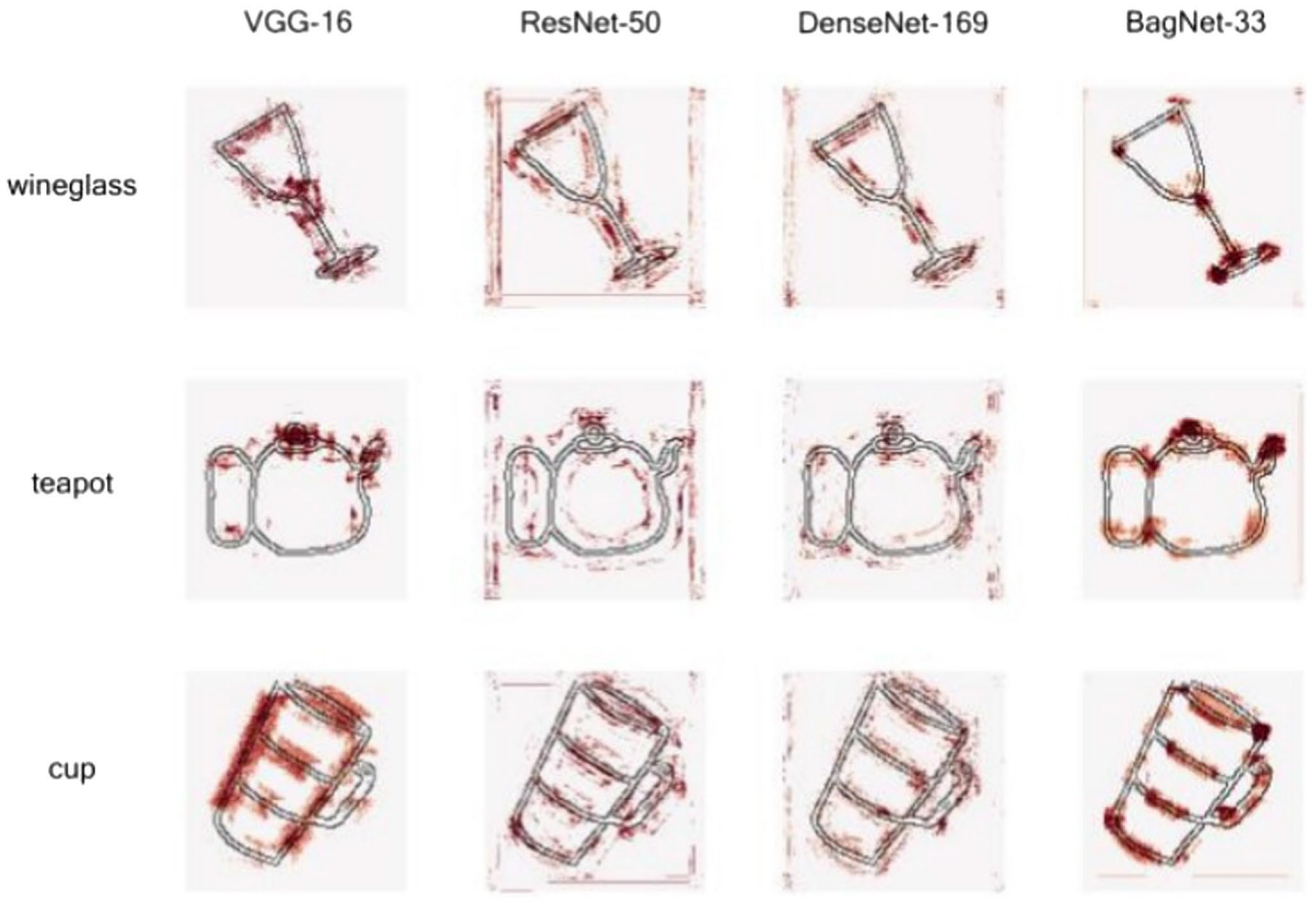
CAM of three objects by different models from Theodorus et al. (2020). The more the dots are clustered, the more of the corresponding area is considered in the model. Note that *VGG-16* and *BagNet-33* learned the representation of object parts. Reprinted with permission.

Bag-of-features (BoF) is a computer vision algorithm that aims to extract the occurrence count of features, and is more interpretable than CNNs. Brendel and Bethge (2019) developed *BagNet*, a neural network that combines the flexibility of CNNs and the interpretability of BoF. Although *BagNet* was originally created to analyze natural images, Theodorus et al. (2020) used this model to interpret sketch classification. They compared *BagNet* to non-interpretable CNNs such as *VGG* (Simonyan and Zisserman, 2015), *ResNet* (He et al., 2016), and *DenseNet* (Huang et al., 2017), to determine whether the increased interpretability is due to the model itself or to the difference between natural images and sketches. *BagNet* was trained to classify sketches into 251 object categories. For each model (*VGG*, *ResNet*, *DenseNet*), the authors extracted and compared class activation maps (CAMs; Zhou et al., 2016) for multiple images. For a given category, CAM indicates which region of an image influences the prediction of that category the most (Figure 4). To go beyond the qualitative interpretation allowed by a simple description of CAMs, Theodorus et al. (2020) developed a quantitative metric of interpretability, the heatmap interpretability (HI) score, which evaluates the quality of a CAM. A high HI score indicates a meaningful heatmap. Figure 4 illustrates that the CAMs from *ResNet-50* and *DenseNet-109* have a low HI compared to *VGG-16* and *BagNet-33*, because highly activated pixels are largely scattered. Concurrently, a questionnaire was used to empirically evaluate the interpretability of the model. Each respondent was given one correctly predicted image per category with its corresponding heatmap and was asked to label object parts according to the heatmap. Comparing the CAMs for several categories, the authors concluded that their model did not use the same features as humans do for classifying object sketches. For example, the CAMs for the categories of ‘sword’ and ‘knife’ showed that the model only focused on the tip of these objects during classification, while humans also considered the handle and the shape of the blade.

Theodorus et al. (2020) provided an example of how interpretable deep learning models could be used for sketches. Although their model does not understand object representation as humans do (Baker et al., 2018; Jacob et al., 2021), training models on different age classes separately and analyzing the heatmaps of several object categories can help formulate hypotheses about the development of drawing behavior in children. CNNs can be used in conjunction with eye tracking. By using the framework proposed by Theodorus et al. (2020), the dots from CAMs for a given object can be compared to those of humans when classifying an object. Eye-tracking and CNN can also be used for a phylogenetic approach to understand the visualization, understanding, and representation of objects of different apes, for a comparison with young children.

In addition to heatmaps, other techniques offer interpretations, such as perturbation-based models. An example of such a method is ZFNet (Zeiler and Fergus, 2014), where parts of a given image are occluded and replaced by a gray square. Using this method, boxes can be occluded to understand which parts of the image are important for classification. However, it should be noted that the transparency of the prediction must be rigorously studied, as it may not be achievable through local interpretations (Ghassemi et al., 2021).

#### Global interpretation

To understand how information flows in the model, another possibility is to study the global interpretation, such as feature visualization. The first convolutional layer of a CNN extracts basic features, that is, edges and color blobs (Qin et al., 2018), which are easy to visualize and understand, while the deeper layers extract more complex shapes, which can describe parts of objects, entire objects, or complex patterns abstract to human perception (Singer et al., 2020, 2021) showed that photographs and drawings are similarly represented in the early and intermediate layers for networks trained on photographs.

Feature visualization has been widely studied in computer vision (Zeiler and Fergus, 2014; Yosinski et al., 2015; Olah et al., 2017), but few studies have been conducted on sketches and drawings. Young-Min (2019) studied the visual characteristics involved in comic book page classification. First, they designed a model to classify comic book pages between several comic artists. They then investigated visual features using a previously published method (Szegedy et al., 2015), with nine representative neurons for each layer. The results showed that, contrary to photograph classification, the features used by the CNN in classifying drawings of comics were not parts of objects, such as face features, but common artistic patterns (e.g., textures).

Applying these techniques to sketch classification, neural networks can discover new features for several classification problems, such as between very young children and chimpanzees, or even compare the drawing style between different cultures. The hierarchical order of the layer can also be meaningful in understanding the drawing behavior of children at several levels, ranging from a stroke to an object shape construction. For instance, when looking for interspecific and intraspecific differences in drawings, the first convolutional layer of a CNN can extract basic features, differentiating humans from other great apes. For a given CNN trained for classification, one can test whether the depth of the layer discriminant of the classifier is linked to the degree of behavioral divergence (that could be developmental, cultural, genetic, or phylogenetic). One would expect early layers to be discriminant enough to classify between species, and deeper layers would be needed for more complex classification, such as cultural or developmental divergences. As orangutans are more dexterous with their hands than chimpanzees and gorillas (Mackinnon, 1974), the first layer could separate humans and orangutans as well as the two other species.

Another way to interpret CNNs is by using the model parameters proposed by Chen et al. (2016), who developed InfoGAN, a generative model for interpretation that maximizes mutual information to discover latent features. This method has been evaluated using various datasets, such as the MNIST dataset (LeCun, 1998), a database of handwritten digits. In this case, the generator was able to discover latent features describing, for example, digit type, width, and rotation of the digits. InfoGAN has also been used on the CelebA face dataset (Liu et al., 2015), revealing encoded features like the azimuth, the presence of glasses, hairstyle, and emotion. From these results, we anticipate that InfoGAN would have a high appeal in studying sketches, to explore the development of perception and representation in children by identifying features that are common and those that are discriminant between children and adult drawings.

Long et al. (2018) collected drawings from young children, older children, and adults to understand how object representation develops with age. They used a method called transfer learning, where a model trained on one task is reused for another task. Transfer learning saves computing resources and allows for high performance with a relatively small number of datasets because it exploits the fact that some properties learned by a model to solve one task are useful for many other related tasks. In the study by Long et al. (2018), sketches were encoded by a pre-trained CNN; features were extracted from layers across several depths; and representational dissimilarity matrices (RDMs) were calculated for each of the three-age classes and compared. Their study showed that the way older children represent objects is more similar to that of adults than young children. Moreover, this also raises the possibility of studying different levels of representation of drawings through different layers of CNNs. Thus, local and global interpretations are possible with CNNs.

## Available datasets for drawing(s) analysis

As a result of the widespread availability of touch-screen devices, drawings and in particular, sketches, can now be more easily collected and analyzed. Moreover, scholars can also collect drawings online through crowdsourcing or online drawing games. However, these datasets have been rarely used in psychological or anthropological studies, possibly because of the lack of associated metadata on the participants, such as their age, location, gender, culture, or drawing skill level. This metadata can be difficult to collect because they may require ethical approval.

Datasets can be organized into two families: amateur and expert datasets. In this review, amateur datasets mostly collate data on sketches and drawings without associated metadata on the person who did the drawing (in particular the drawing skills). Expert datasets gather drawings that have been extracted from books or comics. This kind of data can lead to other difficulties, such as copyrights. Moreover, the style difference between two experts may be significantly larger than that of between two amateurs, meaning that results obtained with one expert dataset may not be easily generalized to another expert dataset. We provide a non-exhaustive list of drawing datasets that are summarized in Table 2.

**Table 2:** Summary of the presented datasets.

Dataset	# of images	# of categories of drawings	Drawing skill	Type of approach (product or process)	Metadata
*Quick, Draw!*	50 million sketches	345 (objects categories)	Amateur	Both	Country
*TU-Berlin dataset*	20,000 sketches	250 (object categories)	Amateur	Both	Human prediction available for each drawing
*Sketchy dataset*	75,471 sketches of 12,500 photographs	125 (object categories)	Amateur	Both	Drawings paired with a photograph
*Manga109 dataset*	109 Japanese comics with 194 pages each	94 (professional creators)	Expert	Product	Labeled rectangles around frames, faces…
*Hicsonmez et al. dataset (2017)*	6,500 pages	24 (professional illustrators)	Expert	Product	

All these datasets are at least partially available online.

### Amateur datasets

A major –and one of the first – sketch datasets is *QuickDraw* by Google. This dataset includes more than 50 million sketches belonging to over 345 object categories (Jongejan et al., 2016). *QuickDraw* is an online game where participants are asked to draw an object in 20 s, and a network is trained to recognize that object. For each sketch, the category is stored, as well as a Boolean indicating if the category was recognized by the game, the timestamp of the sketch, the country where the drawing was made, and the spatial and temporal data of the strokes. Despite some limitations (e.g., the lack of metadata such as the sex and age of the person who did the drawing), *QuickDraw* is a highly promising tool for investigating cultural differences in drawing-based object representations.

The second important amateur dataset is the TU-Berlin dataset, which provides more than 20,000 sketches of 250 categories of common objects drawn by 1,350 unique participants (Eitz et al., 2012). TU-Berlin sketches were collected *via* Amazon Mechanical Turk (AMT), a crowdsourcing marketplace where requesters hire crowd-workers to perform particular tasks (in our case, drawing an object from a given category). Furthermore, each drawing is associated with a second category, for which other participants are asked to identify the drawn object. The temporal order of the strokes is available for each drawing; however, the personal data on the participants are not available.

A third dataset is the Sketchy database, which consists of 12,500 photographs of 75,471 sketches belonging to 125 object categories (Sangkloy et al., 2016). Each sketch is paired with a photograph, and each photograph is linked to a number from 1 to 5, characterizing the ease of sketching. Temporal data on strokes are available for each sketch. As most of the datasets are constructed by asking the participants to draw a particular object, there may be a large variability with respect to the drawn object and its features. For example, when asked to draw a dog, two participants may think about completely different breeds, which can be undesirable for the analyses. For this reason, datasets containing sketches representing photographs can lead to a decrease in variability, which can be an asset for this type of data.

### Experts’ datasets

Among expert datasets, Manga109 (Fujimoto et al., 2016) provides 109 Japanese comic books drawn by 94 professional creators with each book containing 194 pages on average. These books date from 1970 to 2010 and several genres are illustrated. Each page is annotated with rectangular areas characterizing the position of metadata, such as frames, text, and character (face, body), through software developed for this study.

Hicsonmez et al. (2017) collected more than 6,500 pages from a total of 24 children’s book illustrators, with the goal of recognizing the authors using deep learning.

The list of datasets in this review is not exhaustive, only the main datasets are described. Other sketches datasets exist, such as COAD (Tirkaz et al., 2012), SPG (Li et al., 2018), SketchyScene (Zou et al., 2018).

## Future research framework and perspectives

This review provides an overview on how deep learning has been and could be used to increase our knowledge of drawing behavior. Understanding the ontogeny of drawing behavior has many fundamental applications including, diagnosis of pathologies and understanding perception. However, the classical methods used in psychology or comparative cognition, to analyze drawings, rely on verbalization by the author and the subjective interpretation of the experimenter, which limits the reproducibility of results; one way to overcome this is to use deep learning. Simple classification using deep learning can lead to high accuracy, but the interpretability and reliability of the input are not easy to assess, which is also true for supervised (classification, feature visualization) and unsupervised (InfoGAN) learning. Methods have been developed to interpret these results, such as heatmaps and similarity matrices, that are relevant to sketches. Another approach uses generative modeling (e.g., GANs) to generate drawings, to analyze the generative process, and eventually infer the underlying behavior. However, while drawing ontogeny is known to critically depend on various factors such as culture, age, and sex, the large datasets of drawings and sketches, currently used to train CNN and other AI algorithms, usually lack this kind of information. Thus, it is important to develop new datasets, methods, and criteria to advance our understanding of drawing behavior. A dataset with many ancillary variables could, for example, allow cultural analysis. By unraveling the extraordinary predictive capacity of models and through ongoing research to make these models more transparent, AI will undoubtedly significantly contribute to improving our understanding of the fundamental behavior of drawing, for humans and their relatives.

## Author contributions

BB wrote the manuscript with support and approvement from MP, CS, and JR. All authors contributed to the article and approved the submitted version.

## Funding

This project has received financial support from the CNRS through the MITI interdisciplinary programs and from the University of Strasbourg with an IDEX Exploratory Research program.

## Conflict of interest

The authors declare that the research was conducted in the absence of any commercial or financial relationships that could be construed as a potential conflict of interest.

## Publisher’s note

All claims expressed in this article are solely those of the authors and do not necessarily represent those of their affiliated organizations, or those of the publisher, the editors and the reviewers. Any product that may be evaluated in this article, or claim that may be made by its manufacturer, is not guaranteed or endorsed by the publisher.
